# Analysis of a Clapping Vibration Energy Harvesting System in a Rotating Magnetic Field

**DOI:** 10.3390/s22186916

**Published:** 2022-09-13

**Authors:** Yi-Ren Wang, Chao-Kang Feng, Chin-Han Cheng, Pin-Tung Chen

**Affiliations:** Department of Aerospace Engineering, Tamkang University, Tamsui, New Taipei City 25137, Taiwan

**Keywords:** energy harvester, piezoelectric patch, dimensional analysis

## Abstract

This technical note proposes a clapping vibration energy harvesting system (CVEH system) installed in a rotating system. This device includes a rotating wheel, a drive shaft that rotates the wheel, and a double elastic steel sheet fixed on the drive shaft. One of the free ends of the steel is fixed with a magnet, and the free end of the other elastic steel is fixed with a PZT patch. We also install an array of magnets on the periphery (rim) of the wheel. The rim magnets repulse the magnet on the elastic steel sheet of the transmission shaft, causing the elastic steel to oscillate periodically, and slap the piezoelectric patch installed on the other elastic steel sheet to generate electricity. In this study, the authors’ previous study on the voltage output was improved, and the accurate nonlinear natural frequency of the elastic steel was obtained by the dimensional analysis method. By adjusting the rotation speed of the wheel, the precise frequency was controlled to accurately excite the energy harvesting system and obtain the best output voltage. A simple experiment was also performed to correlate with the theoretical model. The voltage and power output efficiencies of the nonlinear frequency to linear frequency excitation of the CVEH system can reach 15.7% and 33.5%, respectively. This study confirms that the clapping VEH system has practical power generation benefits, and verifies that nonlinear frequencies are more effective than linear frequencies to excite the CVEH system to generate electricity.

## 1. Introduction

Most of the man-made mechanical components vibrate, ranging from aircraft fuselage, structures of bridges across the sea, to micro-mechanical components, and “vibration” is a kind of energy expression. Recovering these energies and then using them in human lives has become an important part of green energy. Among them, most researchers have paid attention to the vibration energy harvester (VEH). This device can collect vibration-generated energy through a specific vehicle, and convert it into electrical energy, and the energy can also be stored and used, which can effectively solve the problem of energy consumption. Roundy et al. [1,2] showed that the vibration energy harvesting system has a very low duty cycle and only needs a small space to generate electricity. Erturk and Inman [3] proposed an analytical solution of piezoelectric devices applied to cantilever beams through the assumption of a nonlinear Euler–Bernoulli beam, and analyzed the parallel and series connection of piezoelectric devices. Harne and Wang [4] sorted out the relevant theories about the bistable energy harvester (BEH), explaining the application of the magnet repulsion and the vibration generated by the piezoelectric device to generate electricity.

Yang and Towfighian [5] combined the bistable vibration energy harvesting system with internal resonance (IR) to make the system generate larger amplitude, thereby generating more energy. In addition, Yang and Towfighian [6] also proposed a concept of a bistable vibration energy harvesting system with elastic energy. They added a spring to the magnet at the free end of the beam to compress and elongate the spring in the axial direction, thereby driving the magnet to obtain more potential energy. After adding the elastic energy in this way, the amplitude of the beam is further increased to obtain more electric energy. Zhou [7] et al. installed a magnet at the end of an elastic metal, and a pair of magnets with different magnetic poles were mounted on the base opposite to the magnet. By controlling the angle of the magnet on the base to excite the elastic metal, a bistable energy harvesting system was formed. Wu et al. [8] proposed an accurate theoretical model of the voltage and displacement parameters based on Lenz’s law. The factors affecting the voltage and displacement parameters in the theoretical model were analyzed. However, the accurate nonlinear system frequency was not provided.

Wang et al. [9] proposed the concept of using slapping force to generate more electrical energy conversion. They used two elastic steel sheets to slap (clap) piezoelectric devices, and verified each one with nonlinear theory and experiments. Wang et al. [9,10] found that slapping the piezoelectric patch can achieve a higher power generation effect. By exciting higher modes frequencies on the VEH system, it can achieve higher power generation benefits. Among them, since the deformation of the elastic steel is extremely large, which has exceeded the range that can be simulated by the linear assumption, it is necessary to use nonlinear theory to analyze the vibration behavior of the beam.

Wang and Chu [11] proposed an energy harvesting system that collects the downward airflow from a helicopter or a multi-axis unmanned rotary-wing aircraft and uses the wind force to drive the magnet installed on a windmill to generate repulsive force on a pair of elastic steel sheets. Their clapping VEH system causes the double elastic steel system to clap each other and vibrate periodically in order to generate more electricity than the traditional energy harvesting system. However, they found that the elastic steel of this system cannot match the beating frequency of the elastic steel when the high-frequency windmill rotates, resulting in poor power conversion efficiency when the airflow velocity is too high. This technical note proposes two improved methods based on the model of Wang and Chu [11]: (1) In [11], it is mentioned that the elastic steel cannot match the frequency of the periodic vibration and the frequency of the magnetic field fluctuation, so there will be irregular slaps. This situation can be solved by simply adjusting the length of the elastic steel. (2) In [11], the excitation frequency of elastic steel is a linear frequency, but for this elastic steel, its deformation has exceeded the linear range, so its nonlinear frequency should be considered. Wang et al. [12] proposed a VEH system with rotational magnetic excitation. Through the rotation of the shaft blade, the intermittent magnetic force between the driving magnet and the tip magnetic mass drives the PZT to vibrate nonlinearly. They found that with two driving magnets and 8 mm radial excitation distances, the proposed system captures energy efficiently. Hassan et al. [13] developed a triboelectric vibration energy harvester under rotational magnetic excitation for wind energy harvesting applications. Similar to the work of Wang and Chu [11] and Wang et al. [12], the triboelectric beam generates electricity using the magnetic impact-induced vibration. A single-degree-of-freedom model is used to simulate the generated electrical power. Ambrozkiewicz et al. [14] considered a nonlinear electromagneto system with the rotational magnetic pendulum for energy harvesting. The modelling of electromagnetic effects in different magnets positions was performed by the finite element method (FEM). The experimental results were verified with numerical simulations. The proposed model for the rotational pendulum was used to prove the broadband frequency effect. Enayati and Asef [15] gave a review and analysis of magnetic energy harvester (MEH) applications in a wireless sensor network (WSN). They also provided a case study for feeding navigational sensors mounted on a rotating wheel of vehicles. Their results demonstrated the applicability of their proposed model in specialized applications. Gunn et al. [16] proved that the rotational vibrations can be converted into relatively small but useful amounts of electrical energy that can power wireless sensors. Their experimentally tested device is demonstrated to power a wireless temperature sensor transmitting data every 2 s for a range of more than 1000 rpm of the shaft rotational speed. He et al. [17] proposed a magnetically excited rotating piezoelectric energy harvester with multiple piezoelectric beams connected in series to generate output voltages in excess of 15 V at frequencies below 13 Hz. These research studies proposed various VEH systems that utilize rotating magnetic fields to excite PZTs. Whether analyzing the weight of the magnet or various applications, the excitation frequency is ultimately used to excite the system. However, none of them discuss which excitation frequency is the best. In the present study, the theoretical nonlinear frequency of the nonlinear beam is obtained by the dimensional analysis method, and the rotational speed of the wheel is adjusted to obtain the precise repulsion force of the rotating magnetic field on the elastic steel.

**Figure 1:**
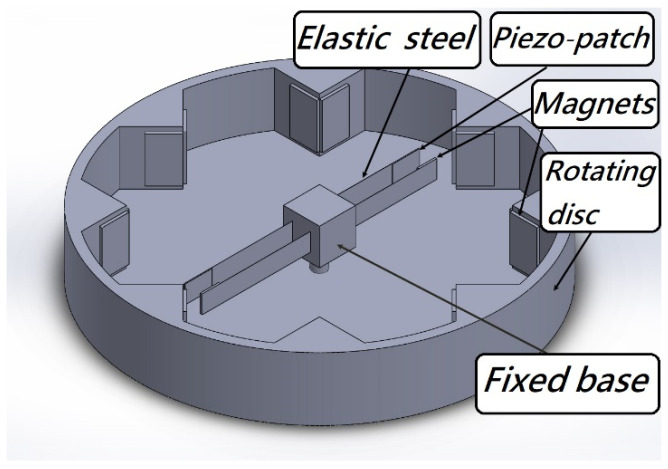
3D schematic diagram of the theoretical model.

**Figure 2:**
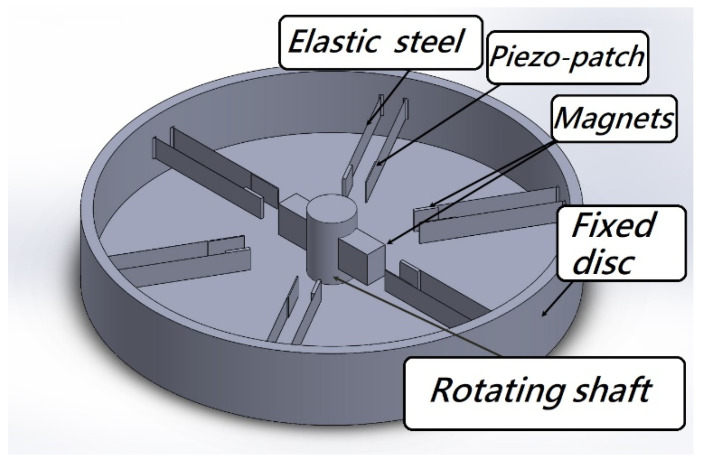
Extended System Design.

The classical VEH system considers the analysis of vibration, but the power generation effects actually depend on the vibration amplitude and the force on the PZT. Therefore, it is a necessary part of the analysis to accurately excite the system and make it generate larger electric energy. In this study, in addition to the establishment and analysis of the VEH nonlinear theoretical model, the correctness of the theory is verified by experiments. The nonlinear system frequencies are derived by dimensional analysis and used to excite the CVEH system. We use the disk to simulate the situation of the rotating wheel (Figure 1), and attach magnets around the disk, so that the magnetic poles can be exchanged when the disk rotates, and then the magnets on the fixed-free beam with a tip mass will be repulsive, so that this beam can be swung periodically by magnetic force. It is worth noting that the device in Figure 1 can also be reversed, as shown in Figure 2, so that each group of double elastic steel systems is fixed on the outer ring of the disc, and the magnet is placed in the middle of the turntable. When the wheel rotates, the steel sheet will also be affected by the magnet, which will cause the steel sheet to swing and generate electricity. This system can make more groups of PZT generate electricity at the same time, and can also be installed on the transmission shaft of the tail boom of the helicopter. The systems of Figure 1 and Figure 2 are only used to show that the concept of the rotating magnetic field can be applied to these two types of systems. The basic principle they used is to employ a rotating magnetic field to excite two pieces of elastic steel and slap each other to generate more electricity. It is believed that it can produce greater economic benefits in the industry.

**Figure 3:**
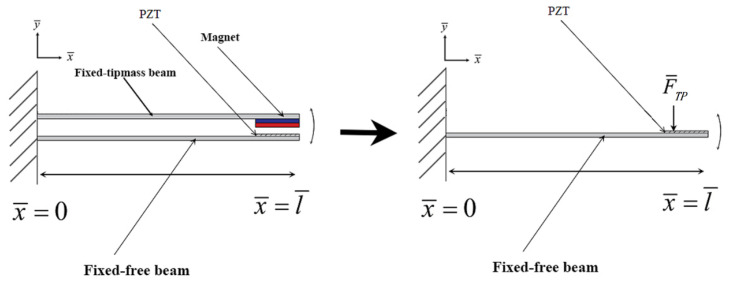
Schematic diagram of the CVEH system.

The theoretical CVEH system is shown in Figure 3, one of which is a fixed-free beam with a tip mass (magnet), and the other is a fixed-free beam with a PZT patch. The CVEH system proposed in this study directly exerts force (${\bar{F}}_{\mathrm{TP}}$) on the piezoelectric device through the effect of slapping, which will enable this design to break through the current bottleneck of energy conversion in the vibration energy harvesting system, and exert a greater function of the VEH system.

## 2. Theoretical Model of Energy Harvesting System

### 2.1. Theoretical Model of Nonlinear Beam

We use the nonlinear beam equation of Wang and Chu [11]:

(1) $$\bar{m}\ddot{\bar{u}}-EA{\bar{u}}^{\prime \prime }=EA(\frac{1}{2}{{\bar{W}}^{\prime }}^{2}-{\bar{u}}^{\prime }{{\bar{W}}^{\prime }}^{2}{)}^{\prime }+EI_{A}{[{\bar{W}}^{\prime }({\bar{W}}^{\prime \prime \prime }-{\bar{u}}^{\prime \prime \prime }{\bar{W}}^{\prime }-2{\bar{u}}^{\prime \prime }{\bar{W}}^{\prime \prime }-3{\bar{u}}^{\prime }{\bar{W}}^{\prime \prime \prime })]}^{\prime }$$

(2) $$\bar{m}\ddot{\bar{W}}-EI{\bar{W}}^{iv}=EA({\bar{u}}^{\prime }{\bar{W}}^{\prime }-{{\bar{u}}^{\prime }}^{2}{\bar{W}}^{\prime }+\frac{1}{2}{{\bar{W}}^{\prime }}^{3}\;{)}^{\prime }+EI[{\bar{u}}^{\prime }{\bar{W}}^{\prime \prime \prime }+{\left({\bar{u}}^{\prime }{\bar{W}}^{\prime }\right)}^{\prime \prime }-\left({{\bar{u}}^{\prime }}^{2}-{{\bar{W}}^{\prime }}^{2}\right){\bar{W}}^{\prime \prime \prime }\;-{\bar{u}}^{\prime }{\left({\bar{u}}^{\prime }{\bar{W}}^{\prime }\right)}^{\prime \prime }-{\left({{\bar{u}}^{\prime }}^{2}{\bar{W}}^{\prime }-\frac{1}{3}{{\bar{W}}^{\prime }}^{3}\right)}^{\prime \prime }\;]{\;}^{\prime }+{\bar{F}}_{TP}$$

**Figure 4:**
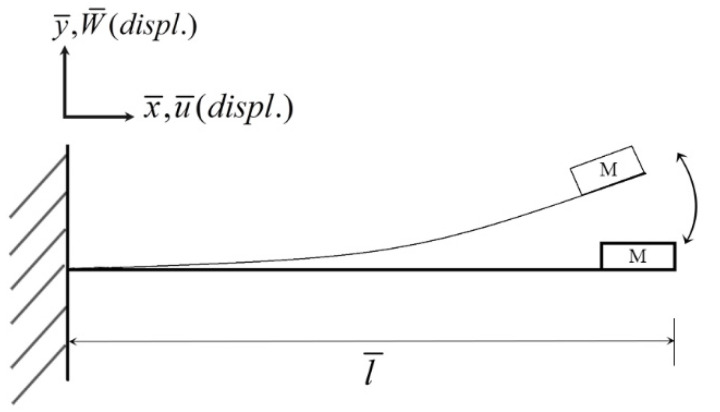
Beam coordinates.

Its coordinates are shown in Figure 4. $\bar{m}$ is the beam mass per unit length, *E* is Young’s modulus, *A* denotes the cross-sectional area of the beam, *I* represents the moment of inertia of the beam, ${\bar{F}}_{TP}$ is the slapping external force on the beam, ${\left(\right)}^{\prime }$ represents the differential with respect to space ($d/d\bar{x}$), $\overset{\cdot }{\left(\right)}$ denotes the time differential ($d/d\bar{t}$), $\bar{u}$ and $\bar{W}$ represent the deformation in the $\bar{x}$ and $\bar{y}$ directions, respectively. The boundary conditions of this elastic beam are:

(3) $$\bar{W}(0,\bar{t})=0,\;{\bar{W}}^{\prime }(0,\bar{t})=0,\;EI_{A}{\bar{W}}^{\prime \prime }(\bar{l},\bar{t})=I_{m}\alpha^{4}a^{4}{\bar{W}}^{\prime }(\bar{l},\bar{t}),\;EI_{A}{\bar{W}}^{\prime \prime \prime }(\bar{l},\bar{t})=-\bar{M}\alpha^{4}a^{4}\bar{W}(\bar{l},\bar{t})$$

In Figure 4, $\bar{M}$ represents the mass of the magnet. In addition, the axial force at the free end of the $\bar{u}$ direction is zero, so the equation of the elastic beam can be simplified to the equation of the simple $\bar{W}$ direction as follows:

(4) $$\bar{m}\ddot{\bar{W}}+\bar{\mu }\dot{\bar{W}}+EI_{A}({\bar{W}}^{\prime \prime \prime }+{\bar{W}}^{\prime }{\bar{W}}^{{\prime \prime }^{2}}+{\bar{W}}^{\prime \prime \prime }{\bar{W}}^{\prime^{2}}{)}^{\prime }=-\frac{1}{2}\bar{m}({\bar{W}}^{\prime }\int_{\bar{l}}^{\bar{x}}\frac{d^{2}}{d{\bar{t}}^{2}}(\int_{0}^{\bar{x}}{\bar{W}}^{\prime^{2}}d\bar{x})d\bar{x}{)}^{\prime }+{\bar{F}}_{TP}$$

in which $\bar{\mu }\dot{\bar{W}}$ is the structural damping,${\bar{W}}^{\prime }{{\bar{W}}^{\prime \prime }}^{2}+{\bar{W}}^{\prime \prime \prime }{{\bar{W}}^{\prime }}^{2}$ represent the nonlinear geometry. $-\frac{1}{2}\bar{m}{\left[{\bar{W}}^{\prime }\int_{\bar{l}}^{\bar{x}}\frac{d^{2}}{d{\bar{t}}^{2}}(\int_{0}^{\bar{x}}{{\bar{W}}^{\prime }}^{2}d\bar{x})d\bar{x}\right]}^{\prime }$ is the shortening effect of the beam.

In order to obtain a wider range of applications and facilitate analysis, we make all theoretical equations dimensionless. With the dimensionless value, we can compare the experimental results of different sizes, and then we only need to convert the theoretical value into dimension according to the dimension of the experiment. It is more convenient for subsequent verification. The dimensionless equation of Equation (4) can be expressed as follows:

(5) $$\overset{**}{W}+\mu \overset{*}{W}+W^{iv}+{W^{\prime \prime }}^{3}+W^{iv}{W^{\prime }}^{2}+4W^{\prime }W^{\prime \prime \prime }W^{\prime \prime }=-\frac{1}{2}{\left[W^{\prime }\int_{1}^{x}\left(\int_{0}^{x}{W^{\prime }}^{2}dx\right)**dx\right]}^{\prime }+F_{TP}$$

The dimensionless parameters are defined as: $l=\frac{\bar{l}}{\bar{l}}=1$, $W=\frac{\bar{W}}{\bar{l}}$, $\mu =\frac{{\bar{l}}^{2}}{\sqrt{\bar{m}EI}}\bar{\mu }$, $F_{TP}=\frac{{\bar{F}}_{TP}}{\bar{m}\bar{l}{\bar{\omega }}^{2}}$,$\bar{\omega }=\sqrt{\frac{EI}{\bar{m}{\bar{l}}^{4}}}$, ${\left(\right)}^{\prime }$ = d/dx, ()* = d/dτ,$\tau =\bar{\omega }\bar{t}$. The dimensionless boundary conditions are defined as:

(6) $$W(0,\tau )=0,\;W^{\prime }(0,\tau )=0,\;W^{\prime \prime }(l,\tau )=0,\;W^{\prime \prime \prime }(l,\tau )=-\hat{m}W(l,\tau )$$

in which $\hat{m}=\frac{\bar{M}}{\bar{l}\bar{m}}$ and is the mass ratio of the magnet mass to the elastic beam.

### 2.2. Theoretical Model of Piezoelectric Patch

The dimensionless Coulomb force of the PZT acting on the nonlinear beam can be obtained from Wang and Chu [11] and is written as:

(7) $$\frac{C_{f}^{2}(\int_{a}^{b}W^{\prime \prime }dx)\nu }{\bar{l}\bar{m}{\bar{\omega }}^{2}}=\eta^{2}(\int_{a}^{b}W^{\prime \prime }dx)\nu =-\frac{\hat{k}\eta^{2}}{e^{R_{P}\tau }}\int_{a}^{b}W^{\prime \prime }dx\int_{0}^{\tau }(\int_{a}^{b}\left(W^{\prime \prime }\right)*dx)e^{R_{P}\tau }d\tau$$

where $\eta^{2}=\frac{C_{f}^{2}}{\bar{l}\bar{m}{\bar{\omega }}^{2}}$, $v=\frac{V}{C_{f}}$, $R_{P}=\frac{1}{{\bar{R}}_{p}C_{p}\bar{\omega }}$, $\hat{k}=\frac{eh_{p}t_{h}}{C_{p}C_{f}}$, ()*=d/dτ, ${\left(\right)}^{\prime }=d/dx$, *a* and *b* are the positions of the piezoelectric patch from one end to the other end, and *C_f_* is the piezoelectric coupling coefficient. The dimensionless voltage (v) can be written as:

(8) $$\nu =-\frac{\hat{k}}{e^{R_{P}\tau }}\int_{0}^{\tau }(\int_{a}^{b}\left(W^{\prime \prime }\right)*dx)e^{R_{P}\tau }d\tau$$

### 2.3. Theoretical Model of Magneto-Electric Equation

The magneto-electric Lorentz force acting on the nonlinear elastic beam is written as ${\bar{C}}_{G}\bar{I}$, where ${\bar{C}}_{G}$ is the electromagnetic coupling coefficient, and $\bar{I}$ is current. The dimensionless equation can be obtained from Wang and Chu [11]:

(9) $$i*+R_{M}i+W*=0$$

The dimensionless current is:

(10) $$i=-\frac{1}{e^{R_{M}\tau }}\int_{0}^{\tau }W*e^{R_{M}\tau }d\tau$$

where $R_{M}=\frac{{\bar{R}}_{M}}{{\bar{C}}_{M}\bar{\omega }}$, ${\bar{C}}_{M}$ is the inductance. We regard the voltage and magnetic force generated by this piezoelectric patch and the magnet as external force terms and add them to the original nonlinear beam motion Equation (5); we can then get the equation of motion integrating magnetoelectricity and piezoelectricity can be obtained as follows:

(11) $$\begin{array}{c} W**+W^{iv}+\mu W*+{W^{\prime \prime }}^{3}+W^{iv}{W^{\prime }}^{2}+4W^{\prime }W^{\prime \prime \prime }W^{\prime \prime }-\frac{\hat{k}\eta^{2}}{e^{R_{P}\tau }}\int_{a}^{b}W^{\prime \prime }dx\int_{0}^{\tau }{\left(\int_{a}^{b}\left(W^{\prime \prime }\right)*dx\right)}^{}e^{R_{p}\tau }d\tau \\ +C_{G}\frac{1}{e^{R_{M}\tau }}\int_{0}^{\tau }W*e^{R_{M}\tau }d\tau =-\frac{1}{2}(W^{\prime }\int_{1}^{x}\left(\int_{0}^{x}{W^{\prime }}^{2}dx\right)**dx{)}^{\prime }+F_{TP} \end{array}$$

where $C_{G}=\frac{{\bar{C}}_{G}^{2}}{{\bar{C}}_{M}\bar{m}{\bar{\omega }}^{2}}$.

The clap force of the two elastic steel sheets is assumed to be the concentrated force acting on the PZT from the other elastic beam (steel) (${\bar{F}}_{TP}$ in Figure 3). The dimensionless slapping force is $F_{TP}=\frac{{\bar{F}}_{TP}}{\bar{m}{\bar{l}}^{2}{\bar{\omega }}^{2}}$. Combining Equations (8) and (11), the theoretical voltage of each mode can be obtained by the fourth-order Runge–Kutta method. Please refer to Wang and Chu [11] for details.

### 2.4. Dimensional Analysis

The main purpose of this technical note is to improve upon the authors’ previous rotating magnetic field vibration energy harvesting system [11]. Wang and Chu [11] found that exciting the natural frequency of elastic steel does not lead to large amplitudes, so the power generation benefit is not as good as expected. We deduce the possibility that the exciting frequency is not the natural frequency of the system. This study therefore finds the exact nonlinear system frequency in a theoretical way. The subsequent experiments in this technical note also show that this theoretical solution is useful. To the best of the authors’ knowledge, there is no literature that analyzes the problem of exciting a vibrating energy harvesting system at nonlinear frequencies. Therefore, a detailed theoretical analysis is necessary in this study. In addition, in order to understand the applicability of this theoretical solution, in the experimental process, the theoretical frequency is applied to excite the system, and the theory and the experiment can be related. Subsequent results also demonstrate that the proposed method can provide better power generation efficiency for the CVEH system.

Wang and Chu [11] used the Method of Multiple Scales (MOMS) to analyze nonlinear equations. The characteristic equations were obtained as follows:

(12) $$\alpha^{3}(-1-\cos\alpha l\cosh\alpha l)+M(-\sin\alpha l\cosh\alpha l+\cos\alpha l\sinh\alpha l)=0$$

The first three eigenvalues can be found as: 2.1264, 4.7142 and 7.8590. The mode shape of the *n*^th^ harmonic mode is $\phi_{n}(x)=(-\sin\alpha_{n}x-\sinh\alpha_{n}x)$$+\frac{\left(\sin\alpha +\sinh\alpha \right)}{\left(-\cos\alpha -\cosh\alpha \right)}$$(-\cos\alpha_{n}x$$-\cosh\alpha_{n}x)$. However, the natural frequencies obtained from these eigenvalues are the linear system frequency, and there is still some error for the nonlinear system frequency. In this study, the analytical solution of the nonlinear frequency of this nonlinear system is obtained by dimensional analysis.

From Equation (11), ignore the nonlinear term of the multiplication of *W* and the damping term μ, and keep the nonlinear term of the third power, then Equation (11) can be reduced to the following Duffing’s type:

(13) $$W**+\omega_{0}^{2}W+\varepsilon W^{3}=0$$

Considering the time domain response, Equation (13) can be rearranged to the function of time and written as:

(14) $$\ddot{x}+\omega_{0}{}^{2}x+\varepsilon x^{3}(t)=0$$

For the sake of simplicity, we choose *x* and *t* to represent function and variable of *W* in the time domain. Please do not confuse this with previous assumptions for dimension or dimensionless variables. The initial conditions for Equation (14) are:

(15) x(0)=a

(16) $$\dot{x}(0)=0$$

where $\omega_{0}$ is the linear natural frequency, and ε<1.

Let x(t;ε) be expressed as:

(17) $$x(t;\varepsilon )=x_{0}\left(t\right)+\varepsilon x_{1}\left(t\right)+\text{......}$$

Substitute Equation (17) into Equations (14)–(16) to obtain the initial value problem for equations of each order,

(18) $$\varepsilon^{0}\text{:}\;{\ddot{x}}_{0}+\omega_{0}{}^{2}x_{0}(t)=0$$

The initial conditions are:

(19) $$x_{0}(0)=a$$

(20) $${\dot{x}}_{0}(0)=0$$

The solution of order $\varepsilon^{0}$ is,

(21) $$x_{0}(t)=a\cos\omega_{0}t$$

(22) $$\varepsilon^{1}\text{:}\;{\ddot{x}}_{1}+\omega_{0}{}^{2}x_{1}(t)=-x_{0}{}^{3}(t)$$

The initial conditions are:

(23) $$x_{1}(0)=0$$

(24) $${\dot{x}}_{1}(0)=0$$

Substituting Equation (21) into Equation (22) yields:

(25) $${\ddot{x}}_{1}+\omega_{0}x_{1}(t)=-a^{3}\cos^{3}\omega_{0}t=\frac{-a^{3}}{4}(3\cos\omega_{0}t+\cos3\omega_{0}t)$$

The particular solution $x_{1p}(t)$ and homogeneous solution $x_{1h}(t)$ can be found as:

(26) $$x_{1p}(t)=\frac{-3}{8\omega_{0}}a^{3}t\sin\omega_{0}t+\frac{a^{3}}{32\omega_{0}{}^{2}}\cos3\omega_{0}t$$

(27) $$x_{1h}(t)=\frac{-a^{3}}{32\omega_{0}{}^{2}}\cos\omega_{0}t$$

Substitute Equations (26) and (27) into Equation (17) to obtain the first-order approximate solution,

(28) $$x(t;\varepsilon )=a\cos\omega_{0}t+\varepsilon (\frac{-3a^{3}}{8\omega_{0}})[(t\sin\omega_{0}t-(\cos3\omega_{0}t-\cos\omega_{0}t)]$$

By using the Lindstedt–Poincare^’^ (L–P) method to eliminate the secular term to obtain an approximate solution that is consistent and valid in the time domain, we let

(29) τ=ωt

where ω is the nonlinear frequency, and

(30) $$\frac{dx}{dt}=\frac{dx}{d\tau }\frac{d\tau }{dt}=\omega \frac{dx}{dt}$$

(31) $$\frac{d^{2}x}{dt^{2}}=\omega^{2}\frac{d^{2}x}{dt^{2}}$$

Asymptotically expand the frequency ω to the natural frequency $\omega_{0}$ as:

(32) $$\omega (\varepsilon )=\omega_{0}+\varepsilon \omega_{1}+\varepsilon^{2}\omega_{2}+O(\varepsilon^{3})$$

Substituting Equations (31) and (32) into Equations (14) and (16) yields:

(33) $$\omega^{2}\frac{d^{2}x}{dt^{2}}+\omega_{0}{}^{2}x(\tau ;\varepsilon )=-\varepsilon x^{3}$$

The initial conditions are the same as Equations (15) and (16).

The next step is to do perturbation asymptotic expansion of x(τ;ε):

(34) $$x(\tau ;\varepsilon )=x_{o}(\tau )+\varepsilon x_{1}(\tau )+\varepsilon^{2}x_{2}(\tau )+O(\varepsilon^{3})$$

Substitute Equation (34) into Equation (33) to obtain the initial value problem of each order:

(35) $$\varepsilon^{0}\text{:}\;\frac{d^{2}x_{0}}{d\tau^{2}}+x_{0}(\tau )=0$$

The initial conditions are the same as Equations (19) and (20). The solution can be found as:

(36) $$x_{0}(\tau )=a\cos(\tau )$$

(37) $$\varepsilon^{1}\text{:}\;\omega_{0}{}^{2}[\frac{d^{2}x_{1}}{d\tau^{2}}+x_{1}(\tau )]=-x_{0}{}^{3}-2\omega_{0}\omega_{1}\frac{d^{2}x_{0}}{d\tau^{2}}$$

The initial conditions are the same as Equations (23) and (24). Substituting Equation (36) into Equation (37), we get:

(38) $$\frac{d^{2}x_{1}}{d\tau^{2}}+x_{1}(\tau )=\frac{1}{\omega_{0}{}^{2}}(2\omega_{0}\omega_{1}a\cos(\tau )-a^{3}\cos^{3}(\tau ))=\frac{1}{\omega_{0}{}^{2}}[(2\omega_{0}\omega_{1}a-\frac{3}{4}a^{3})\cos(\tau )-\frac{a^{3}}{4}\cos(3\tau )]$$

To eliminate the secular term in Equation (38), we let the secular term be 0:

(39) $$(2\omega_{0}\omega_{1}a-\frac{3}{4}a^{3})=0$$

that is $\omega_{1}=\frac{3}{8\omega_{0}}a^{2}$. The particular solution $x_{1p}(\tau )$ and homogeneous solution $x_{1h}(\tau )$ can be found as,

(40) $$x_{1p}(\tau )=\frac{a^{3}}{32\omega_{0}{}^{2}}\cos(3\tau )$$

(41) $$x_{1h}(\tau )=\frac{-a^{3}}{32\omega_{0}{}^{2}}\cos(\tau )$$

and the $x_{1}(\tau )$ is expressed as:

(42) $$x_{1}(\tau )=x_{1p}(\tau )+x_{1h}(\tau )=\frac{a^{3}}{32\omega_{0}{}^{2}}(\cos(3\tau )-\cos(\tau ))$$

(43) $$\varepsilon^{2}\text{:}\;\omega_{0}{}^{2}[\frac{d^{2}x_{2}}{d\tau^{2}}+x_{2}(\tau )]=-(\omega_{1}{}^{2}+2\omega_{0}\omega_{2})\frac{d^{2}x_{0}}{d\tau^{2}}-3x_{0}{}^{2}x_{1}-2\omega_{0}\omega_{1}\frac{d^{2}x_{1}}{d\tau^{2}}$$

The initial conditions are:

(44) $$x_{2}(0)=0$$

(45) $${\dot{x}}_{2}(0)=0$$

Substituting Equations (34) and (42) into Equation (43) yields:

(46) $$\begin{array}{c} \omega_{0}{}^{2}[\frac{d^{2}x_{2}}{d\tau^{2}}+x_{2}(\tau )]=(\omega_{1}{}^{2}+2\omega_{0}\omega_{2})a\cos(\tau )-\frac{3a^{5}}{32\omega_{0}{}^{2}}\cos^{2}(\tau )(\cos(3\tau )-\cos(\tau ))\\ +\frac{2}{32}a^{3}\frac{\omega_{1}}{\omega_{0}}(9\cos(\tau )-\cos(3\tau )) \end{array}$$

Simplify Equation (46) into:

(47) $$\begin{array}{c} \frac{d^{2}x_{2}}{d\tau^{2}}+x_{2}(\tau )=\frac{1}{\omega_{0}{}^{2}}(a\omega_{1}{}^{2}+2a\omega_{0}\omega_{2}+\frac{6a^{5}}{128\omega_{0}{}^{2}}-\frac{2a^{3}}{32\omega_{0}}\omega_{1})\cos(\tau )\\ +\frac{48a^{5}}{256\omega_{0}{}^{4}}\cos(3\tau )-\frac{6a^{5}}{256\omega_{0}{}^{4}}\cos(5\tau ) \end{array}$$

To eliminate the secular term in Equation (47), we let the secular term be 0:

(48) $$\omega_{2}=\frac{1}{2a\omega_{0}}(\frac{2a^{3}}{32\omega_{0}}\omega_{1}-a\omega_{1}{}^{2}-\frac{6a^{5}}{128\omega_{0}{}^{2}})$$

where $\omega_{1}=\frac{3}{8\omega_{0}}a^{2}$, and then $\omega_{2}=\frac{1}{2a\omega_{0}}(\frac{6-36-12}{256\omega_{0}{}^{2}})a^{5}=\frac{-21a^{4}}{256\omega_{0}{}^{3}}$. Finally, the second-order approximate solution of ω(ε) is obtained as:

(49) $$\omega (\varepsilon )=\omega_{0}+\varepsilon \omega_{1}+\varepsilon^{2}\omega_{2}+\ldots =\omega_{0}+\frac{3}{8}\frac{a^{2}}{\omega_{0}}\varepsilon -\frac{21a^{4}}{256\omega_{0}{}^{3}}\varepsilon^{2}+O(\varepsilon^{3})$$

or

(50) $$\frac{\omega }{\omega_{0}}=1+\frac{3}{8}\frac{a^{2}}{\omega_{0}{}^{2}}\varepsilon -\frac{21a^{4}}{256\omega_{0}{}^{4}}\varepsilon^{2}+O(\varepsilon^{3})$$

When the secular term is eliminated in Equation (47), it can be reduced into:

(51) $$\frac{d^{2}x_{2}}{d\tau^{2}}+x_{2}(\tau )=\frac{{48a}^{5}}{256\omega_{0}{}^{4}}\cos(3\tau )-\frac{6a^{5}}{256\omega_{0}{}^{4}}\cos(5\tau )$$

The particular solution $x_{2p}(\tau )$ and homogeneous solution $x_{2h}(\tau )$ of Equation (51) can be found as:

(52) $$x_{2p}(\tau )=\frac{a^{5}}{1024\omega_{0}{}^{4}}\cos(5\tau )-\frac{24a^{5}}{1024\omega_{0}{}^{4}}\cos(3\tau )$$

(53) $$x_{2h}(\tau )=\frac{-a^{5}}{1024\omega_{0}{}^{4}}\cos(\tau )+\frac{24a^{5}}{1024\omega_{0}{}^{4}}\cos(\tau )$$

The solution for $x_{2}(\tau )$ is:

(54) $$x_{2}(\tau )=x_{2p}(\tau )+x_{2h}(\tau )=\frac{a^{5}}{1024\omega_{0}{}^{4}}\cos(5\tau )-\frac{24a^{5}}{1024\omega_{0}{}^{4}}\cos(3\tau )+\frac{23a^{5}}{1024\omega_{0}{}^{4}}\cos(\tau )$$

Finally, from Equation (34), the consistent and effective second-order approximate solution of Duffing’s nonlinear equation in the time domain is obtained as:

(55) $$x(\tau ;\varepsilon )=a\cos(\tau )+\varepsilon \frac{a^{3}}{32\omega_{0}{}^{2}}(\cos(3\tau )-\cos(\tau ))+\varepsilon^{2}\frac{a^{5}}{1024\omega_{0}{}^{2}}(\cos(5\tau )-24\cos(3\tau )\;+23\cos(\tau ))+O(\varepsilon^{3})$$

### 2.5. Numerical Simulation

**Figure 5:**
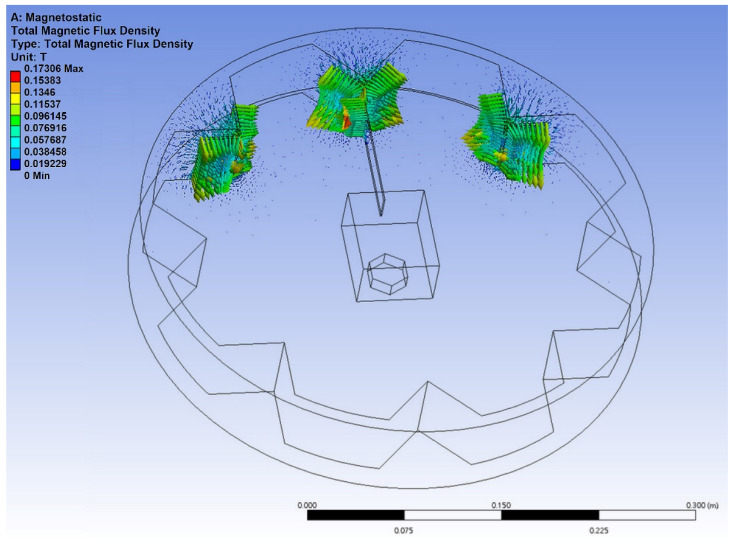
Distribution of magnetic flux.

**Figure 6:**
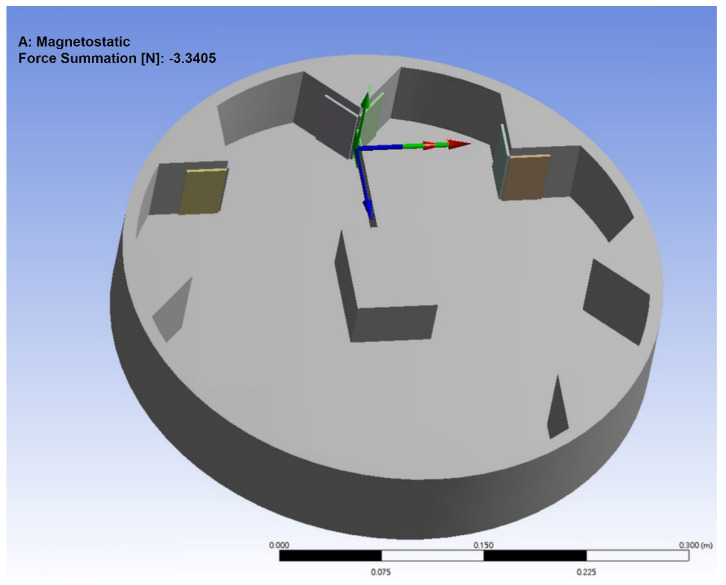
The sum of the force of the magnet.

**Figure 7:**
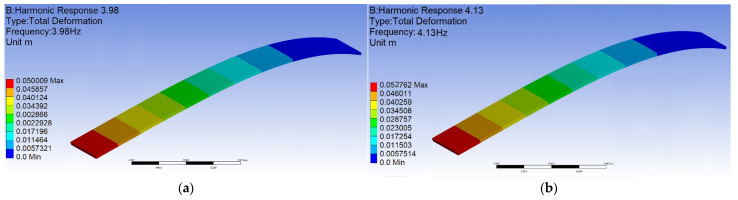
Displacement of the 10 cm elastic steel (**a**) excited by linear frequency, (**b**) excited by nonlinear frequency.

In order to further understand the magnetic field distribution of this device and the mutual repulsive force between the magnets, and to observe the displacement of the elastic steel, to preliminarily estimate the possible deformation of the PZT, we used the commercial software ANSYS to construct this model. We focus on the magnetic field between the magnets at the three key positions on the rim of the wheel and the magnet at the end of the elastic steel. The purpose of the numerical simulation is to analyze the magnetic flux of the elastic steel with lengths of 10 cm, 7.5 cm and 5 cm and the force of the magnets at the end point of the elastic steel. Taking a 10 cm elastic steel sheet as an example, Figure 5 shows the distribution of magnetic flux, and the maximum value can reach 0.173 Wb. Figure 6 shows the force of the magnet at the end of the elastic steel, and the sum is 3.34 N. The simulation results show that the design of this model works. From Equation (55) and converting to the dimension of this system, we used the linear frequency (3.98 Hz) and the nonlinear frequency (4.13 Hz) as the excitation frequency of the external force to analyze the displacement of the elastic steel. Figure 7a,b shows the results for linear frequency and nonlinear frequency excitation, which are 5.00 and 5.27 cm, respectively. Similarly, when the elastic steel sheets of 7.5 cm and 5 cm are excited at these two frequencies, the displacements are 1.99 cm, 2.11 cm (for 7.5 cm steel) and 0.50 cm, and 0.55 cm (for 5 cm steel), respectively. Among them, we found that the steel displacements excited by the nonlinear frequencies are larger, and the longer the elastic steel, the larger its end point displacement. Although a longer elastic steel may theoretically have a larger power generation, the authors’ previous analysis showed that a longer elastic steel cannot have a larger power generation in this clapping VEH system. The reason is that the repulsive force of the magnet and the elastic restoring force of the elastic steel cannot be perfectly matched when the slap force is considered. Therefore, longer elastic steel does not mean better power generation efficiency. These phenomena may not be simulated by a simple FEM model, so we use experiments to analyze their power conversion effects.

## 3. Experiment Setup

### 3.1. Design of the Experimental Device

**Figure 8:**
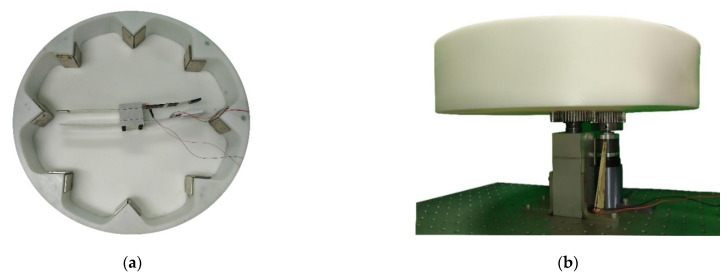
Experimental setup: (**a**) top view, (**b**) side view.

We combined the rotating disk of the experimental device and a gear with a bolt, with a variable speed motor and bearing, fixed on the optical table (Figure 8). Eight sets of magnets are mounted around the rotating disk. When the rotating disk rotates, the magnetic field can be changed. By changing the magnetic field, the magnets on the elastic steel are attracted and repelled, and then the elastic steel is driven to swing. The PZT patch installed on the elastic steel generates electrical energy by the deformation and slap force from the other elastic steel. From Equation (12), the first two eigenvalues can be found as 2.1264 and 4.7142. After conversion to the dimension of this system (a 7.5 cm elastic steel sheet), the linear natural frequencies of the first two modes are 3.98 Hz and 19.56 Hz, respectively. Since the wheel is equipped with eight sets of magnets, each time the rotating disk rotates one cycle, the impact on the elastic steel is eight cycles, and the rotational speed corresponding to the linear natural frequencies of the first two modes is about 30 RPM and 146 RPM. From Equation (55), and choosing *a* = 2 cm, ε = 0.4, the nonlinear natural frequencies of the first two modes are 4.13 Hz and 19.60 Hz, respectively. The rotational speed corresponding to the first two modes of the nonlinear natural frequencies of the elastic steel is about 31 RPM and 147 RPM. We use the variable speed motor to control the rotational speed of the rotating disk to excite the natural frequency of the elastic steel to make it reach the maximum amplitude. We have experimentally measured the displacement of the elastic steel subjected to a magnetic force. When the rotational speed of the wheel is 30 RPM (3.98 Hz), the displacement of the elastic steel tip is about 4.8 cm (numerical prediction is 5 cm, see Section 2.4)), and at 31 RPM (4.13 Hz), the displacement of the steel tip is about 5.0 cm (numerical prediction is 5.27 cm, see Section 2.4)). The mass of the magnet is about 35 g, and the mass of the elastic steel is about 10 g. We used the parametric data of the magnets attached by the manufacturer and simulated the actual situation with ANSYS. For a magnet with a mass of 35 g and a displacement of about 5.0 cm for this elastic steel, 3.34 N is reasonable. Using the magnetic force simulated by ANSYS, the displacement of the elastic steel after being subjected to force is also the same as the experiment, so the verification is correct. Then, we can measure the output voltage through the built-in function of the IMC^©^ system (System Access, Taipei, Taiwan).

### 3.2. Voltage Output

**Figure 9:**
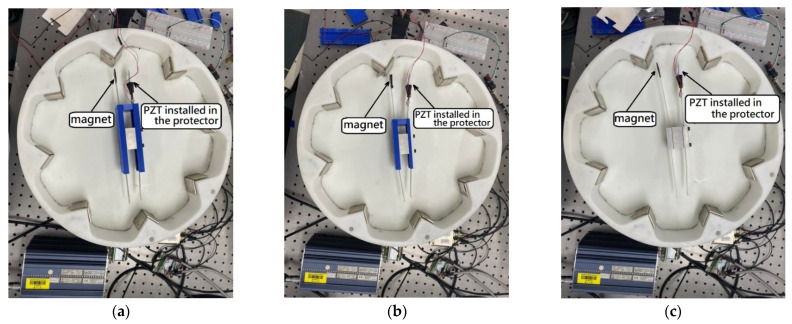
Elastic steel length: (**a**) 5 cm, (**b**) 7.5 cm, (**c**) 10 cm.

**Figure 10:**
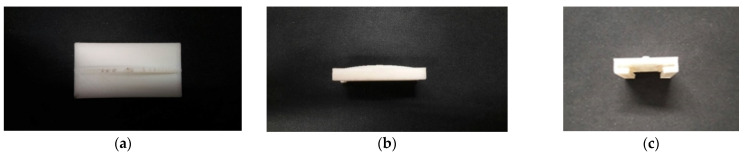
PZT protector: (**a**) top view, (**b**) side view, (**c**) front view.

In the experiment of Wang and Chu [11], in order to improve the power generation efficiency, elastic steel with a longer length (10 cm) is used, but when the modes above the 2nd mode are excited, irregular oscillation occurs, resulting in a great decrease in the power generation efficiency. We speculate that the speed of the modes above the second mode may be faster. After the elastic steel that is too long is ejected due to the magnetic force, the elastic restoring force is not enough to pull the elastic steel back, such that the magnetic force and elastic force of the whole system cannot match with each other. This technical note uses experiments to measure the power generation efficiency of another two sets of elastic steel sheets with lengths of 7.5 cm and 5 cm, respectively, to determine the benefit of this CVEH. The combination of the three elastic steels and PZT is shown in Figure 9. In addition, in order to reduce the damage of the slap force on the PZT, we designed a PZT protector to insert into the PZT, as shown in Figure 10. The purpose is to ensure that the slap force can be fixed on a specific position of the piezoelectric patch, and to avoid irregular slap points and slap force, resulting in uneven force on the piezoelectric patch, or affecting the frequency of periodic vibration.

**Figure 11:**
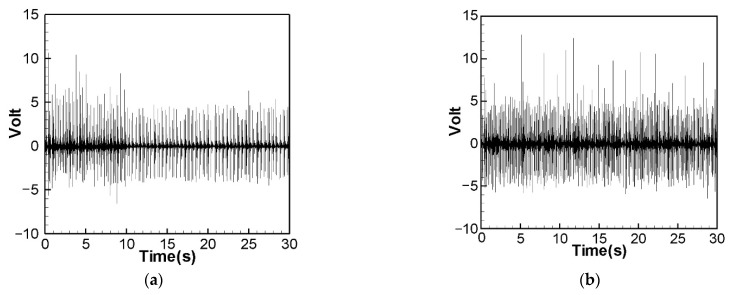
Experimental voltage output for nonlinear frequency excitation (elastic steel 5 cm). (**a**) 1st mode, (**b**) 2nd mode.

**Figure 12:**
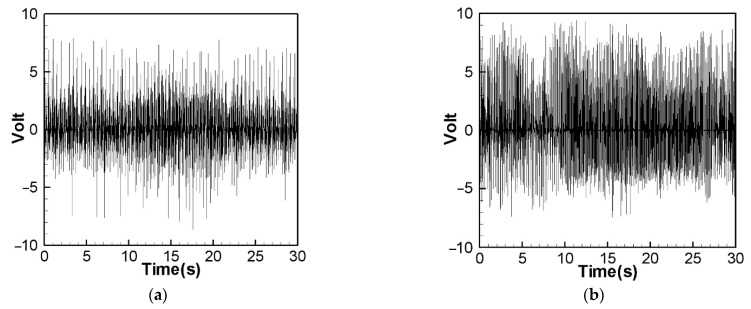
Experimental voltage output for nonlinear frequency excitation (elastic steel 7.5 cm). (**a**) 1st mode, (**b**) 2nd mode.

**Figure 13:**
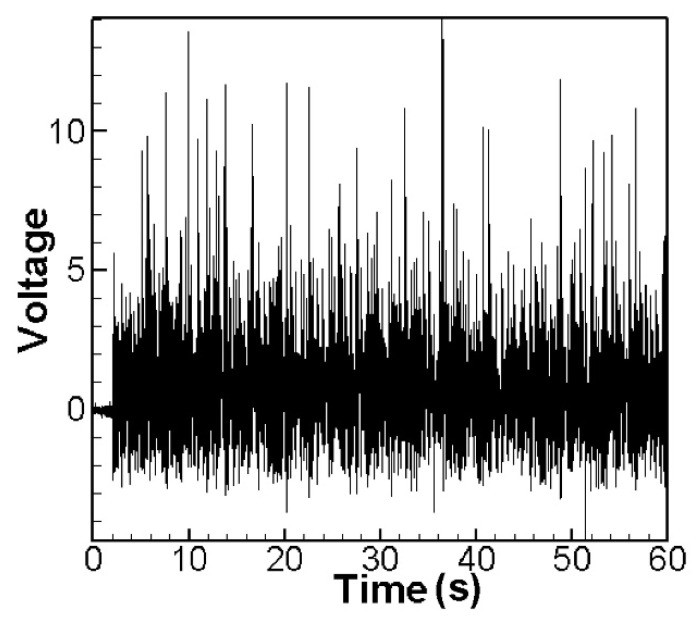
Experimental voltage output for nonlinear frequency excitation (elastic steel 10 cm), 1st mode.

We excited the CVEH system with the rotational speed corresponding to the nonlinear natural frequency, and the voltage outputs of the elastic steel sheets with lengths of 5 cm, 7.5 cm and 10 cm are shown in Figure 11, Figure 12 and Figure 13. The time-voltage charts shown in this technical note are the system voltage output records captured when the system is stable. The horizontal axis time unit is second. These output voltage charts show the power conversion efficiency of the system. We chose to display the results in terms of time and voltage because the system is designed to be used in a mechanically rotating system. The effect of the power conversion of this system can be observed over time. Figure 11 shows the 1st and 2nd modes’ voltage output diagrams of the 5 cm elastic steel sheet (PZT length = 4.5 cm) excited by the nonlinear natural frequency. The average value of the root mean square of the 1st mode is about 1.8906 V. Figure 12 shows the 1st and 2nd modes’ voltage output diagrams of a 7.5 cm elastic steel sheet excited by nonlinear natural frequency; the average value of the 1st mode root mean square is about 2.0374 V. Since the vibration amplitudes of the 7.5 cm elastic steel sheet are higher than 5 cm, the power generation efficiency is higher than 5 cm.

It is worth noting that Figure 13 is the 1st mode voltage output diagram of a 10 cm elastic steel sheet excited by nonlinear natural frequency. Since the elastic steel sheet that is too long is ejected due to the magnetic force, the elastic restoring force is not enough to pull the elastic steel sheet back. The magnetic force and elastic force of the whole system cannot match each other. Therefore, the output voltage of the 2nd mode cannot be measured. This situation is the same as the conclusion of Wang and Chu [11]. However, using the 7.5 cm elastic steel sheet, we successfully extended the power generation benefit to the 2nd mode. We found that choosing the appropriate length of elastic steel (7.5 cm) is helpful for the power generation efficiency. Therefore, the following experiments and theoretical verifications are based on the 7.5 cm elastic steel sheet. In this experiment, two energy harvesting systems will be studied, namely: a linear frequency excited CVEH system, and a nonlinear frequency excited CVEH system. The experimental measurements of the voltage output of the first 2 modes of the CVEH system are compared with the theoretical predictions.

## 4. Discussion

According to the current equation (Equation (8)), the following voltage function can be obtained:

(56) $$V=-{\bar{R}}_{p}\int_{a}^{b}eh_{p}t_{h}{\dot{\bar{W}}}^{\prime \prime }d\bar{x}$$

Based on the length of the piezoelectric patch, and the length of the elastic steel sheet, we substitute the MKS unit into Equation (56) for calculation, and use the fourth-order Runge–Kutta method to solve Equation (11), and obtain the theoretical value of the voltage output. The voltage outputs excited by the linear frequency and the nonlinear frequency in each mode are plotted respectively, and then the root mean square is taken. The theoretical root mean square is compared with the experimental root mean square to confirm the feasibility of this model.

**Figure 14:**
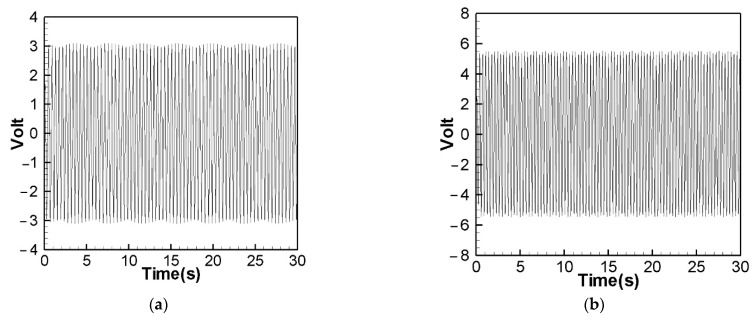
Theoretical voltage prediction for “linear” frequency excitation. (**a**) 1st mode, (**b**) 2nd mode.

**Figure 15:**
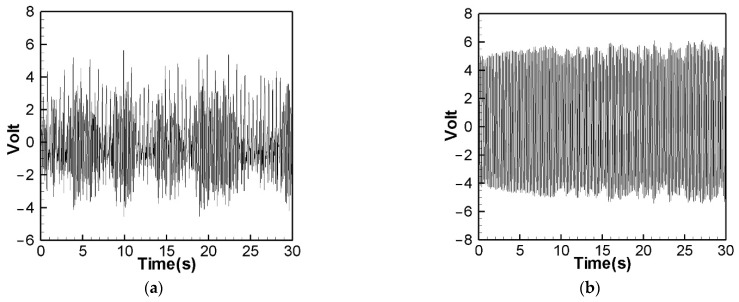
Experimental voltage output for “linear” frequency excitation. (**a**) 1st mode, (**b**) 2nd mode.

**Figure 16:**
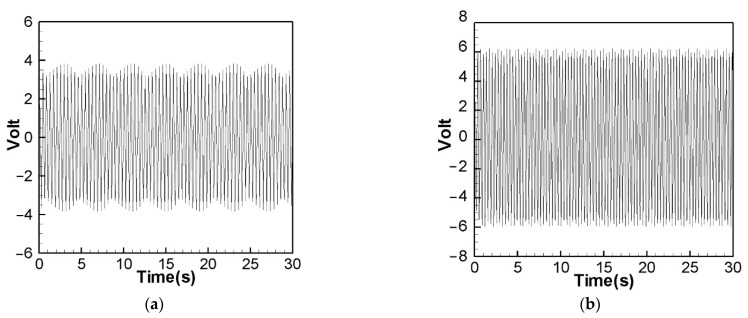
Theoretical voltage prediction for “nonlinear” frequency excitation. (**a**) 1st mode, (**b**) 2nd mode.

**Figure 17:**
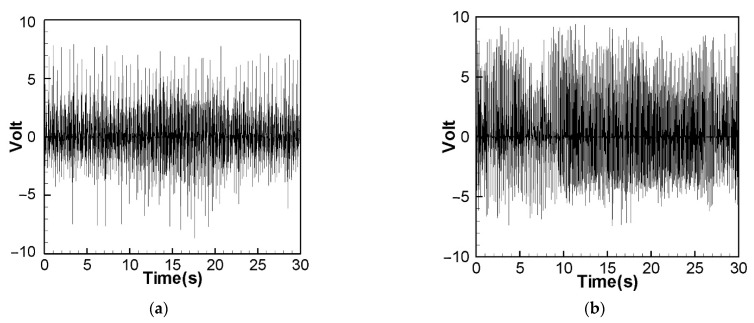
Experimental voltage output for “nonlinear” frequency excitation. (**a**) 1st mode, (**b**) 2nd mode.

Figure 14 is the theoretical output voltage generated by the 1st and 2nd modes of the “linear frequency” of the CVEH system after substituting the dimensional value and solved by the RK-4 method. Figure 15 is the experimental voltage generated by the “linear frequency” of the CVEH 1st and 2nd modes. Figure 16 is the theoretical output voltage generated by the 1st and 2nd modes of the “nonlinear frequency” of the CVEH system after substituting the dimensional value and solved by the RK-4 method. Figure 17 is the experimental voltage generated by the “nonlinear frequency” of the CVEH 1st and 2nd modes. The results are compared in Table 1 and Table 2.

According to Figure 14a, the theoretical voltage of the 1st mode is between +3 V and −3 V, and its RMS (root mean square) value is 1.8866 V. According to Figure 15a, the RMS value obtained from the experiment is 1.8208 V, the error is 3.61%. The theoretical voltages, experimental output voltages, and relative errors of the other mode are shown in Table 1. Figure 16a shows the theoretical voltage outputs generated by the “nonlinear frequency” of the first mode of the CVEH system. Figure 17a shows the experimental voltage outputs of the 1st mode generated by the “nonlinear frequency”. According to Figure 16a, the theoretical voltage of the first mode is between +4 V and −4 V, and its RMS value is 2.1143 V, which is different from the experimental value (Figure 17a), which is 2.1074 V, the error is 3.26% (Table 2). The voltage outputs of the 2nd mode can be found in Figure 14b, Figure 15b, Figure 16b, Figure 17b. The results and errors can be found in Table 1 and Table 2. Compared with the error of the 1st and 2nd modes of Wang and Chu [11] (6~9.2%), the method proposed in this technical note (errors are of 3.26~4.9%) can obtain excellent results. The power outputs of the linear and nonlinear frequency excitations of this CVEH are listed in Table 1 and Table 2, respectively. For the Mode 1 case, the power output efficiency of nonlinear frequency to linear frequency excitation is from 0.29678 mW to 0.2223 mW, an increase of 33.5%. For the Mode 2 case, the power output efficiency of nonlinear frequency to linear frequency excitation is from 1.0168 mW to 0.8103 mW, an increase of 25.3%. We also compare the efficiency in voltage output between elastic steels of different modes for linear and nonlinear frequency inputs. According to the experimental results from Table 1 and Table 2, the efficiency of nonlinear frequency excitation to linear frequency and for Mode 1 and Mode 2 is to increase by 15.7% and 6.4%, respectively.

Looking at the above, no matter what kind of system it is, the higher the mode, the better the power generation efficiency, and exciting the system’s nonlinear frequency obviously makes the amplitude larger, so it has a better power generation effect than the linear frequency excited CVEH system.

## 5. Conclusions

This research takes the fixed-free beam with a tip mass as the main frame model to analyze its vibration mode and energy harvesting system. Based on the model of Wang and Chu [11], this technical note proposes two improved methods: (1) By adjusting the length of the elastic steel sheet (7.5 cm) to solve the problem of irregular slaps; (2) In this study, the theoretical nonlinear frequency of the system was obtained by the dimensional analysis method. Adjust the rotating speed of the wheel to obtain the precise excitation frequency for the elastic steel. Better power generation efficiency can be obtained.

Compared with the 1st and 2nd modes of Wang and Chu [11], the error is about 6~9.2%. Based on the method proposed in this technical note, the error of its 1st and 2nd modes is about 3.26~4.9%, and excellent results can be obtained.

The output voltage of the high mode is higher than the output voltage of the low mode. The CVEH system proposed in this study, through the effect of clapping, directly exerts force on the piezoelectric patch, which can be applied to the shafts of automobiles or motorcycles, and can also be used as the transmission shaft in the tail boom of a helicopter, which is of great application value. Furthermore, the purpose of this note is to suggest improvements to the authors’ previous research. This study also provides two improvement methods which are “theoretical nonlinear frequency function” and “the proper elastic steel length”. In particular, the theoretical solution of nonlinear frequencies is a result that no one has proposed so far. This originality is believed to be enough to contribute to the field of VEH research. This kind of CVEH design can break through the energy conversion bottleneck of the vibration system of the single piezoelectric patch of the current vibration energy harvesting system, and can play a greater function of the VEH system.

## Figures and Tables

**Table 1:** Theoretical, experimental and error for the first 2 modes of linear frequency excitation.

Lnr Frq.	Mode 1	Mode 2
Theo. (V)	1.8866	3.7851
Expt. (V)	1.8208	3.4863
Expt. (mW)	0.2223	0.8103
Error (V)	3.61%	8.57%

**Table 2:** Theoretical, experimental and error for the first 2 modes of nonlinear frequency excitation.

Nlnr Frq.	Mode 1	Mode 2
Theo. (V)	2.1143	3.89
Expt. (V)	2.1074	3.7084
Expt. (mW)	0.29678	1.0168
Error (V)	3.26%	4.90%
